# The EpiQuant Framework for Computing Epidemiological Concordance of Microbial Subtyping Data

**DOI:** 10.1128/JCM.01945-16

**Published:** 2017-04-25

**Authors:** Benjamin M. Hetman, Steven K. Mutschall, James E. Thomas, Victor P. J. Gannon, Clifford G. Clark, Frank Pollari, Eduardo N. Taboada

**Affiliations:** a Department of Biological Sciences, University of Lethbridge, Lethbridge, Alberta, Canada; b National Microbiology Laboratory at Lethbridge, Public Health Agency of Canada, Lethbridge, Alberta, Canada; c National Microbiology Laboratory at Winnipeg, Public Health Agency of Canada, Winnipeg, Manitoba, Canada; d Centre for Foodborne, Environmental and Zoonotic Infectious Diseases, Public Health Agency of Canada, Guelph, Ontario, Canada; UNC Health Care System

**Keywords:** Campylobacter jejuni, ecological epidemiology, epidemiological concordance, molecular epidemiology, molecular subtyping, sampling metadata, whole-genome sequencing

## Abstract

A fundamental assumption in the use and interpretation of microbial subtyping results for public health investigations is that isolates that appear to be related based on molecular subtyping data are expected to share commonalities with respect to their origin, history, and distribution. Critically, there is currently no approach for systematically assessing the underlying epidemiology of subtyping results. Our aim was to develop a method for directly quantifying the similarity between bacterial isolates using basic sampling metadata and to develop a framework for computing the epidemiological concordance of microbial typing results. We have developed an analytical model that summarizes the similarity of bacterial isolates using basic parameters typically provided in sampling records, using a novel framework (EpiQuant) developed in the R environment for statistical computing. We have applied the EpiQuant framework to a data set comprising 654 isolates of the enteric pathogen Campylobacter jejuni from Canadian surveillance data in order to examine the epidemiological concordance of clusters obtained by using two leading C. jejuni subtyping methods. The EpiQuant framework can be used to directly quantify the similarity of bacterial isolates based on basic sample metadata. These results can then be used to assess the concordance between microbial epidemiological and molecular data, facilitating the objective assessment of subtyping method performance and paving the way for the improved application of molecular subtyping data in investigations of infectious disease.

## INTRODUCTION

The analysis of pathogens through the application of techniques adapted from molecular biology has become an essential part of many modern epidemiological investigations (i.e., “molecular epidemiology”) targeted at the prevention and control of infectious diseases and improving our understanding of how infectious disease agents circulate between/within natural reservoirs and affected populations (1, 2). Molecular subtyping of bacteria allows differentiation between closely related isolates of the same species and can be instrumental in determining if an isolate forms part of an epidemiologically linked cluster. However, an ongoing challenge in molecular epidemiology has been the effective interpretation of subtyping data. While subtyping results connect isolates into groups related by molecular or phenotypic criteria (i.e., clusters), the extent to which these clusters correspond to the underlying epidemiology of the pathogen is not generally known.

Assessment of the epidemiological relevance of isolates sharing a molecular subtype has typically been carried out manually, based on the aims of the analysis. Clusters of genetically or phenotypically related isolates are produced by using one or more molecular subtyping methods, and relevant epidemiological attributes, such as membership in an outbreak group, are superimposed and subjected to interpretation on a cluster-by-cluster basis, with additional context such as subtype reproducibility, subtype prevalence, and subtype variability in the organism also being considered (3–5). While this general approach represents a pragmatic solution to the need for interpretation criteria based on epidemiological relevance, it lacks the systematic rigor required to comprehensively assess subtyping results and their concordance with underlying characteristics related to the ecology and epidemiology of the bacterial isolates in question. In light of the important role of molecular typing in public health investigations, it becomes necessary to develop analytical approaches to systematically assess this relationship.

In this study, we present a model for computing the similarity between bacterial isolates based on attributes commonly documented within isolate sampling records (e.g., source, time, and geography of sampling) and the development of a framework for assessing the concordance between the “epidemiologic signal” of bacterial isolates and their subtyping data. We assess the utility of this framework on a data set of 654 isolates of the important zoonotic pathogen Campylobacter jejuni sampled from across Canada and demonstrate how the model can be used to (i) quantify the epidemiological similarity between C. jejuni isolates, (ii) assess the relative abilities of subtyping methods to cluster isolates into cohesive epidemiologically linked groups, and (iii) identify subtype clusters with significantly increased specificity for the underlying epidemiology of bacterial isolates, facilitating targeted epidemiological investigations.

## RESULTS

### Development of a model for computing source similarities using C. jejuni isolates from the Canadian Campylobacter Comparative Genomic Fingerprinting Database.

Sources for comparison were selected by using available sampling information from the Canadian Campylobacter Comparative Genomic Fingerprinting Database (C3GFdb), a repository containing curated metadata on over 22,000 Campylobacter isolates for which the granularity has been kept largely consistent, simplifying the process of identifying nonredundant sources (*n* = 40) to test our method for computing source similarities.

**FIG 1:**
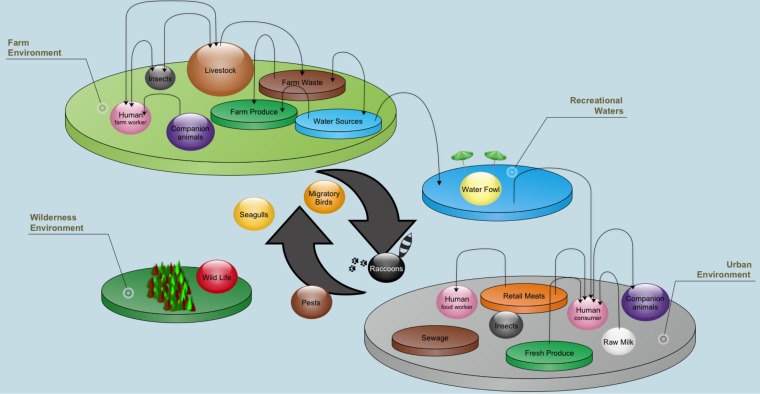
Conceptual framework outlining major environments and interactions in the C. jejuni transmission chain. The model incorporates all C. jejuni sampling sources in the C3GFdb used in the analysis of source distances. Arrows indicate either unidirectional or bidirectional flow of C. jejuni throughout the “farm-to-fork” continuum. Sources not located on one of the four “ecological islands” are considered transitory and have high exposure to multiple environments.

**FIG 2:**
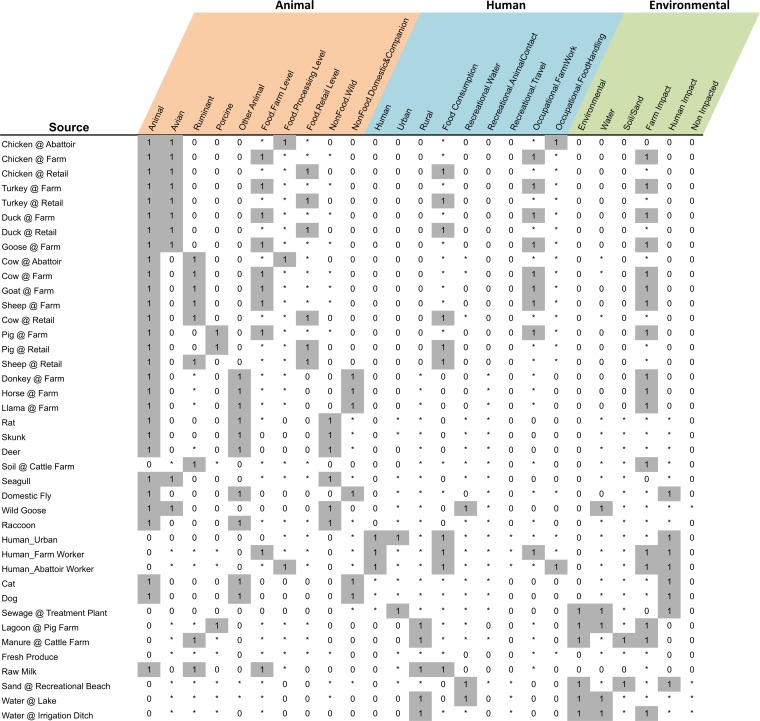
Example of the “epidemiological rubric” used to assess source comparisons. The epidemiological profile for each of 40 unique sources from the C3GFdb was determined independently based on a set of 25 epidemiological attributes derived from the conceptual framework presented in Fig. 1. The character state of “1” is used to indicate a strong association, “*” is used to indicate a partial association, and “0” is used to indicate little or no association with the attribute indicated in each column, where the status reflects the perceived strength of the relationship based on the user's knowledge. The distance between any two sources was computed by comparing their respective epidemiological profiles, with the pairwise source similarity based on the number of matching and partially matching epidemiological attributes as a proportion of the total number of attributes examined (*n* = 25).

Developing a rubric for comparing Campylobacter sampling sources from the C3GFdb involved describing the epidemiological profile of each source using a series of attributes constructed from a conceptual framework that outlined major environments and interactions that we believe are important for the C. jejuni transmission chain (Fig. 1). Each source was then assessed independently against these attributes, and the distance between any two sources was computed by comparing their respective epidemiological profiles, with the pairwise source similarity being based on the number of matching and partially matching epidemiological attributes as a proportion of the total number of attributes examined (*n* = 25). An example of the rubric used to assess the unique source identifiers against epidemiologically relevant attributes is shown in Fig. 2.

**FIG 3:**
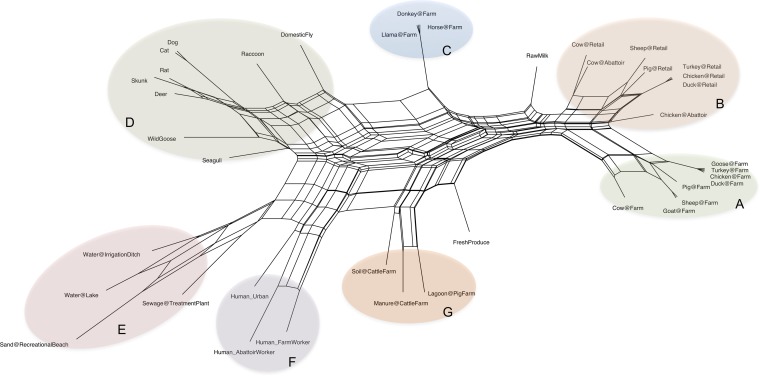
Neighbor network split graph based on C. jejuni source distances. The split network was calculated based on the model for source distances (Δ*s*) in the model. Clusters A to G are highlighted to show the clustering of highly similar sources into the same regions of the graph. Distances were calculated in R by using the phangorn package, and a split graph was then plotted by using Splitstree software, using the equal-angle method.

Pairwise comparison of the epidemiological profiles derived for each source using our rubric resulted in a matrix summarizing the overall “source distance” between all sources used in this study. We constructed a neighbor-network split graph (Fig. 3) based on the source distances in order to confirm whether the resulting source matrix was congruent with our conceptual representation of Campylobacter transmission networks. Clustering results from the split graph demonstrated significant agreement with those proposed in our original conceptual framework. For example, entries related to farm food animal sources—food animals (cluster A) and meat products and abattoir samples (cluster B)—grouped in the same area of the network, and these entries grouped separately from farm-based companion animals (cluster C) and a group comprised of domestic companion animals and wild animals straddling the urban-rural environment (cluster D). A separate region of the network included groups directly related to environmental and human inputs (clusters E and F). A group of farm animal-related environmental sources (cluster G) was found to group midway between the environmental-water-related sources in cluster E and farm animal sources in cluster A, consistent with the dual nature of the source input. While major groupings were readily identified by the split graph shown in Fig. 3, a considerable amount of reticulation, or splits, was observed, and this is consistent with shared characteristics between sources not derived from the same principal headings (i.e., “human,” “animal,” or “environmental”) used to construct the rubric shown in Fig. 2.

**FIG 4:**
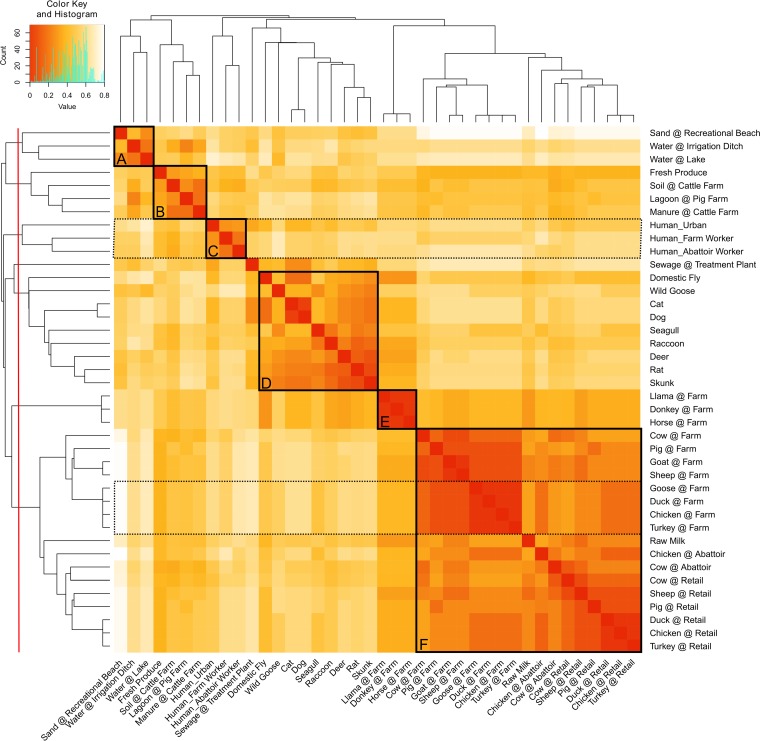
All-versus-all heat map depicting pairwise similarities between C. jejuni sources included in this study (*n* = 40). Source similarities were given by the formula 1 − Δ*s*. Darker shading indicates stronger similarity between sources. Groups A to F represent memberships created at a clustering threshold of 50% (red line at the left). Dotted black boxes are used as a visual aid in the analysis to highlight secondary similarities observed off the “self-versus-self” axis. The heat map was created in R by using custom scripts and the heatmap.2 function from the Gplots package.

To further examine the effect of shared epidemiological attributes on the overall pairwise source comparison, we constructed a hierarchically clustered heat map illustrating the similarity between all pairwise sources (Fig. 4). This visualization yielded several epidemiologically relevant groupings consistent with those observed in Fig. 3; at the same time, areas of similarity away from the 45° (i.e., “self-versus-self”) axis in Fig. 4 reflect epidemiological relationships that lie outside the major groupings outlined in the split graph analysis. An example of this can be seen within cluster F, which is comprised of food animal sources from farm through to retail levels. A subgroup of farm-based poultry sources (i.e., goose, duck, chicken, and turkey) within this cluster displays high secondary similarity to other on-farm food animal sources (i.e., cow, pig, goat, and sheep) and to poultry sources at the abattoir and retail levels. Results from the source model can also be seen to delineate between similar sources that differ at a small number of attributes based on differences in likely primary exposures to C. jejuni. For example, of the three human sources in cluster C, the “Human_Urban” source exhibits higher similarity to animals with urban exposure (e.g., companion animals, raccoons, seagulls, and deer) and retail food sources, the “Human_Farm Workers” source demonstrates higher similarity to on-farm food animals, and the “Human_Abattoir Workers” source expresses strong similarity to abattoir- and retail-based animal sources.

### Combining components to compute epidemiological distance.

**FIG 5:**
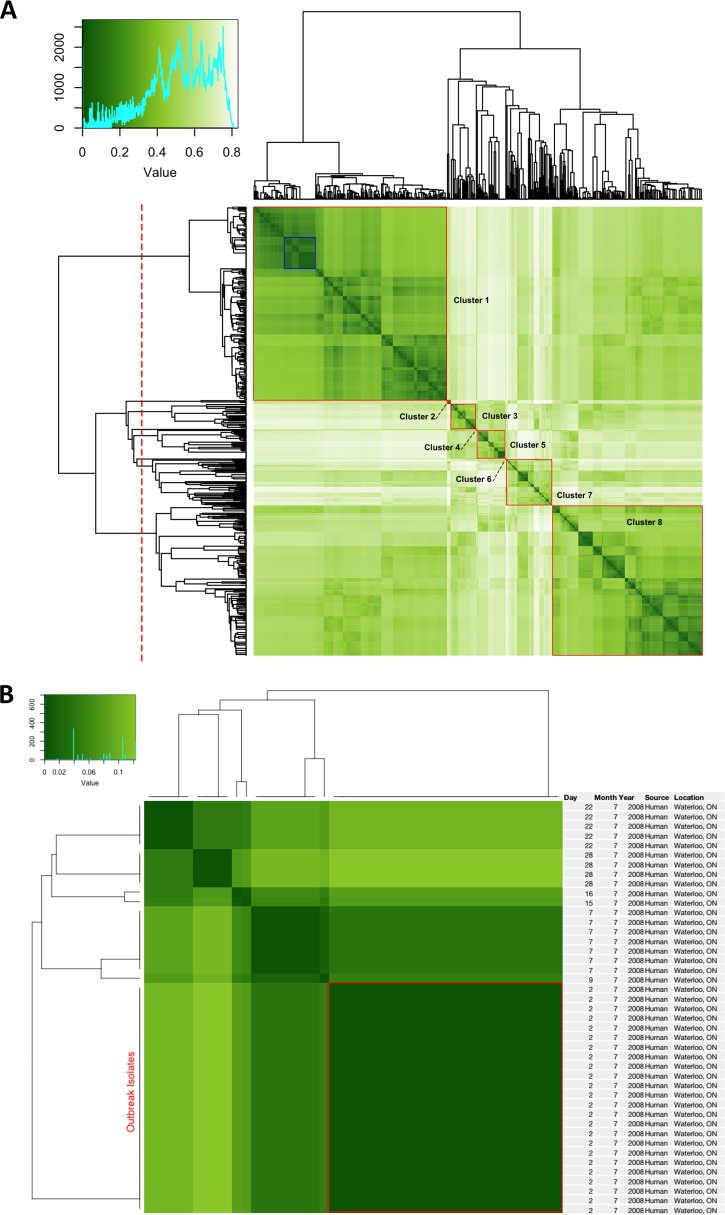
Hierarchical clustering of C. jejuni isolates based on total epidemiological distance (Δε) computed by using the EpiQuant framework. (A) Clustering of the complete set of isolates used in this study (*n* = 654). Darker shading indicates higher similarity between isolates based on the comparison of their sampling metadata (source, temporal, and geospatial) and the Δε calculation outlined in equation 3 using source, temporal, and geospatial coefficients of 0.5, 0.3, and 0.2, respectively. A histogram displaying the frequency of pairwise Δε values observed, ranging from 0 (completely similar) to 1 (completely dissimilar), is shown (top left). (B) Human clinical isolates collected in the same municipality within a 4-week period, including those from a confirmed campylobacteriosis outbreak (also highlighted in blue in panel A), are shown along with their basic metadata. The heat map was generated in R by using the heatmap.2 function from the Gplots package. Clustering of the resulting distances was done by using the “single-linkage” algorithm, and clusters were identified at the 50% threshold (dotted red line at the left).

An example of the total epidemiological distance (Δε) for all isolates in our data set (*n* = 654) was derived from the application of our model for calculating Δε (i.e., see equation 3, below) using sample metadata and is presented in Fig. 5. In combining the source distances described above with geographic positioning data (GPS) and collection dates using weighting ratios of 50%, 30%, and 20% for the σ, τ, and γ coefficients, respectively, a pairwise matrix describing the total epidemiological distance of all isolates from our data set of 654 C. jejuni isolates was created. The adjustable coefficients γ, τ, and σ are used for assigning weights to each component based on *a priori* epidemiological considerations. For example, a bacterial species known to be highly source restricted may then require a higher value for σ to provide additional weight to the source relative to the geospatial and temporal variables to account for the increased significance when observing a difference in the source.

In general, the groups that resulted from clustering based on Δε represented cohesive epidemiological units comprised of bacterial isolates from similar source, temporal, and geospatial cohorts.

For example, cluster 1 comprised 282 human clinical isolates of C. jejuni from Ontario, Canada, with further subclustering based on distances between sampling dates, ranging from January 2006 to November 2008. Within this cluster is a subset of 43 human clinical isolates collected during a 4-week period in the summer of 2007 (Fig. 5A, highlighted in blue). These isolates also include a set of 24 isolates that were confirmed epidemiologically to belong to an outbreak cluster. As shown in Fig. 5B, the outbreak isolates share identical temporal, location, and sampling source data and thus cluster together with an average epidemiological similarity (1 − Δε) value of 1. Isolates collected within the same municipality and in a similar time frame that were not part of the outbreak are shown to cluster separately, with epidemiological similarities ranging from 0.87 to 0.99. Cluster 2 included isolates derived from raccoon sources in Ontario across a narrow sampling time (October 2011 to July 2012). Cluster 3 comprised farm-based food animal isolates sampled from various locations across Alberta, Canada, in 2004 to 2006. Cluster 5 contained isolates sampled from animal sources at both the farm and retail levels, with subclusters being delimited by their source and sampling locations (e.g., “Chicken@Retail” samples from British Columbia, Canada, and “Cow@Farm” samples from Alberta, Canada) as well as the sampling dates, which ranged from 2009 to 2012. Cluster 7 included isolates sampled from environmental sources (e.g., “Water@Irrigation Ditch”), and this is consistent with results from the pairwise source analysis (Fig. 2 and 3), which suggests that environmental sources form a distinct group separate from animal and human sources. Cluster 8 comprised most of the food animal-related isolates in the data set: all isolates contained within this cluster were derived from retail or farm animal sources and encompass a close geographic range in Ontario, Canada.

As was observed with the pairwise source similarity matrix (Fig. 4), a considerable amount of secondary similarity can be seen off the 45° axis due to partial similarity across some, but not all, components. For example, cluster 1 (human) and cluster 8 (food animal) share significant secondary similarity due to the shared geospatial and temporal components of the isolate subsets.

### Use of Δε to assess the epidemiological concordance of subtyping methods.

We wished to investigate the use of the epidemiological similarity (i.e., 1 − Δε) between two isolates estimated by our framework as a means to quantify the epidemiological concordance of subtyping methods. Using multilocus sequence typing (MLST) and comparative genomic fingerprinting (CGF) data from our collection of 654 C. jejuni isolates, we computed the epidemiological cluster cohesion (ECC) value, the average pairwise epidemiological similarity for each subtyping cluster in the data set, and compared the ECC values obtained with each method. Furthermore, as CGF has been shown to have greater discriminatory power than MLST (6) and MLST data can be analyzed at two levels of resolution, clonal complex (CC) and sequence type (ST), these data were also used to investigate epidemiological concordance as a function of a method's discriminatory power (Table 1).

**TABLE 1:** Typing statistics for methods calculated from the current data set of C. jejuni isolates (*n* = 654)

Method	No. of clusters	SID^*a*^	Adjusted Wallace value		
			CGF	ST	CC
CGF	183	0.982		0.671	0.880
ST	179	0.950	0.233		0.999
CC	66	0.887	0.126	0.412	

a SID, Simpson's index of diversity.

**FIG 6:**
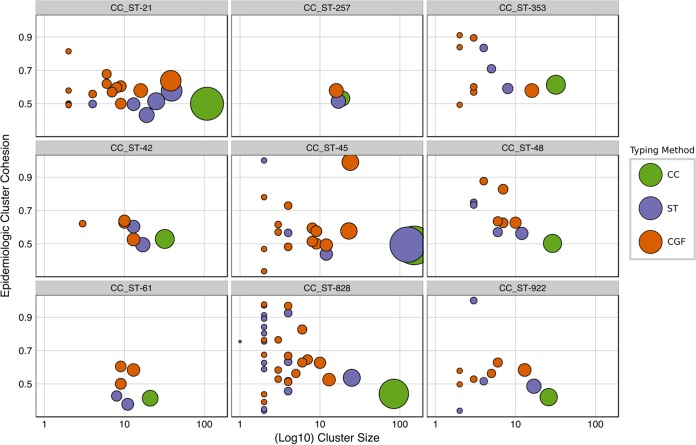
Comparison of ECC values for clusters generated via MLST CCs, MLST STs, and CGF for the isolates used in this study (*n* = 654). Individual facets of the plot contain the isolates from each of nine dominant MLST clonal complexes in the data set (indicated at the top of each box). Green circles denote “parent clusters” based on CCs; membership in subclusters is also shown. The relative membership size of each cluster is indicated by the radius of each circle on the plot and given by the values on the *x* axis. The average ECC of each cluster is given by its position along the *y* axis. Subclusters containing only one member, which were excluded from the ECC analysis, were excluded from the figure (ST, *n* = 101; CGF, *n* = 62). The plot was generated in R using the ggplot2 package.

Compared to the average ECC values of isolates not belonging to clusters (0.471 ± 0.165), we observed that each subtyping method assembled isolates into clusters with high average ECC values (*P* < 0.001) and that higher-resolution methods resulted in increased overall ECC values. The lower-resolution subtyping method (i.e., CC) assembled isolates into larger clusters with a lower overall ECC value (0.486 ± 0.183) than those of higher-resolution methods (i.e., MLST and CGF), which generated several smaller clusters from the CC assignments, and these had higher overall ECC values (0.505 ± 0.197 for ST and 0.543 ± 0.223 for CGF; *P* < 0.001), which is consistent with the increased epidemiological concordance of clusters obtained with the higher-resolution methods. To illustrate this observation, isolates from the nine largest CCs in our data set (*n* = 516) are presented in Fig. 6, with each subplot illustrating a single CC and its splitting into several smaller ST and CGF subtyping clusters that tend to exhibit higher ECC values than those of the original parent cluster.

### Adjustment of Δε parameters to identify subtyping clusters with differing epidemiological characteristics.

To demonstrate the flexibility of the EpiQuant model for assessing the epidemiological cohesion of subtyping clusters based on the differential weighting of geospatial, temporal, and source parameters, we computed Δε for all isolates in the data set based on two additional sets of inputs for γ, τ, and σ coefficients. The first iteration favored relationships based on source relationships (e.g., 80% source, 10% temporal, and 10% geospatial weightings), and the second iteration emphasized temporal associations (e.g., 10% source, 80% temporal, and 10% geospatial weightings). Combined with the original Δε results shown in Fig. 5, we then applied these data to compute the ECC values of CGF subtypes in our data set in an attempt to identify clusters that were highly source or temporally specific.

**FIG 7:**
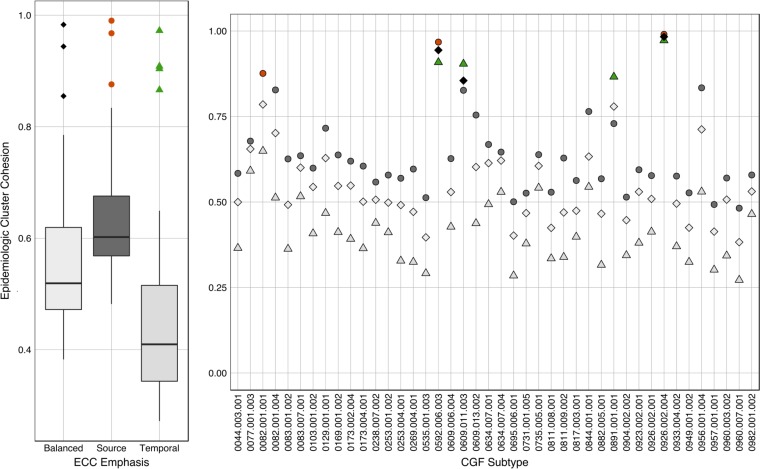
Epidemiological cluster cohesion analysis of CGF clusters using adjusted source, temporal, and geospatial coefficients. (Left) Box plots of the total distribution of ECC values for CGF clusters containing >3 isolates when calculating the Δε value using different sets of source/temporal/geospatial coefficients, including 50:30:20 (i.e., balanced) (left), 80:10:10 (i.e., source emphasis) (middle), and 10:80:10 (i.e., temporal emphasis) (right) ratios. The box bounds the IQR divided by the median, and whiskers extend to a maximum of 1.5 IQR above and below each box. Outliers were identified by using Tukey's method and are indicated as orange circles (source emphasis) or green triangles (temporal emphasis) above the plots. (Right) Distribution of ECC values for individual CGF clusters. ECC values based on “balanced coefficients” (light gray diamonds), “source emphasis” (dark gray circles), and temporal emphasis (light gray triangles) are shown. Black diamonds, orange circles, and green triangles represent outliers identified from the box plot analysis in panel A.

Results from the ECC analysis of CGF subtyping data reveal differences in the distributions of ECC values observed for CGF clusters based on the input coefficients used (Fig. 7A). The ECC distributions show that favoring temporal interactions results in a significantly lower average ECC value (0.458 ± 0.173) than those calculated with a greater emphasis on source relationships (0.640 ± 0.124; *P* < 0.001) or when the “balanced” coefficient set was used (0.564 ± 0.141; *P* = 0.003). This observation is consistent with the wide distribution of temporal signals in the data set (i.e., sampling years from 2004 to 2012). No significant difference was observed between the overall ECC values achieved when “source” versus balanced approaches were compared.

Outliers were identified with significant source and temporal associations based on all three sets of coefficients. To confirm whether the ECC results were indeed reflecting highly biased temporal or source associations, we examined the metadata for each of these outlier subtypes (Table 2). For example, subtype “0082.001.001” was associated with a single source type (Human_Urban), yielding a high ECC value when assessed by favoring the source component despite a temporal range spanning 7 months. In contrast, subtypes 0609.011.003 and 0891.001.001 were identified as being highly specific temporally due to short sampling periods (8 and 17 days, respectively) and had high ECC values when assessed by favoring the temporal component despite being associated with multiple sources. Subtypes 0592.006.003 and 0926.002.004 were identified as being both highly temporal and source specific based on high ECC values obtained under both sets of coefficients. The metadata for both of these clusters revealed a single sampling source collected within a narrow window of time (e.g., Pig@Farm with a 14-day sampling period and Human_Urban with a 10-day time period, respectively).

**TABLE 2:** Metadata summary of clusters identified as statistical outliers in the ECC box plot analysis in Fig. 7

Subtype	Outlier type(s)	Source range(s)	Temporal range (mo/day/yr)
0082.001.001	Source	Human_Urban	01/29/2008–08/28/2008
0592.006.003	Source, temporal	Pig@Farm	03/22/2005–04/05/2005
0609.011.003	Temporal	Chicken@Retail, Cow@Farm	06/04/2007–06/12/2007
0891.001.001	Temporal	Chicken@Retail, Human_Urban, Pig@Retail	07/06/2007–07/23/2007
0926.002.004	Source, temporal	Human_Urban	06/26/2008–07/07/2008

## DISCUSSION

Molecular subtyping techniques have become an essential part of modern epidemiological investigations of infectious disease. Subtyping data have been used to identify outbreaks and their vehicles of transmission (7–13), to study the dynamics of pathogen circulation throughout natural reservoirs (14–16), and to assess the population structure of bacterial disease agents, identifying subgroups important to human health (17–19). A consistent feature in the evolution of the field of molecular epidemiology has been the continuing development and refinement of approaches for molecular typing. In general, the drive for novel methods has been motivated by the search for improvements in performance criteria such as discriminatory power and deployability (6, 20, 21) and by the mitigation of problems that can arise when adapting a given subtyping method to a particular pathogen of interest (22–24). Coupled with continuing technical advances in molecular biology, the search for approaches useful for distinguishing and classifying bacterial strains has led to the development of a large number of subtyping methods now available (25, 26).

A significant challenge with the emergence and proliferation of new molecular typing methods has been the lack of systematic approaches for objectively assessing and comparing methods. In 2006, Carriço et al. described a framework for quantitatively assessing different typing systems using performance criteria such as discriminatory power and partition congruence (27). We previously used this framework for comparing the performance of CGF, a novel method for C. jejuni subtyping developed in our group, to that of MLST, the leading method for C. jejuni subtyping (6), and for assessing both methods against the phylogenetic signal in whole-genome sequence (WGS) data (28). This approach has been useful for assessing the concordance of methods against one another, which is particularly useful when comparing a novel method to a well-established “gold standard.” Critically, although subtyping data are used in the context of epidemiological investigations, the epidemiological concordance of subtyping results is an element that has escaped systematic examination.

The “Tenover criteria,” which were introduced over 2 decades ago, have provided guidance on the interpretation of results generated by using pulsed-field gel electrophoresis (PFGE) (29). It is generally acknowledged that subtyping data must be interpreted in the proper epidemiological context (i.e., epidemiological relevance) while taking into consideration additional factors such as the reproducibility of the method with a particular organism, the genotypic variability of the organism being subtyped, the prevalence of the pattern in question, and outbreak characteristics (3). To date, many studies have been performed by comparing the results of molecular typing with epidemiological metadata using manual methods: once genetic relationships between isolates are determined via subtyping, epidemiological data are examined in an attempt to assess whether subtyping clusters are consistent with the underlying epidemiology (16, 18, 30, 31). More recently, visualizations based on mapping color-coded epidemiological metadata onto dendrograms derived from subtyping data have been used to facilitate this assessment (4). While such approaches have been extremely successful for identifying subtyping clusters related to particular epidemiological considerations, a major disadvantage is that they are qualitative and require significant manual interpretation, making them impractical for the systematic examination of large data sets.

In this investigation, we have focused on (i) establishing an approach for summarizing the epidemiological signal in sampling metadata from C. jejuni isolates, (ii) developing a method for computing the epidemiological similarity between pairs of C. jejuni isolates, and (iii) developing a framework for evaluating the epidemiological concordance of subtyping data to compare the performances of two leading methods of C. jejuni subtyping. As a high-priority foodborne pathogen primarily associated with sporadic illnesses and a number of possible sources, C. jejuni poses significant challenges to analyses based on descriptive epidemiological parameters alone (32). Moreover, although temporal and geospatial data figure prominently in epidemiological investigations of C. jejuni, the sampling source is a parameter that has been shown to contribute significantly to genotypic variation (33). As there is currently no means of measuring the similarity of sampling sources, C. jejuni presents an excellent, if complex, model organism with which to establish a model for source-source comparisons.

We first developed a conceptual framework incorporating major routes of transmission for the spread of C. jejuni throughout various sources and vectors in the farm-to-fork continuum. This exercise enabled us to identify basic attributes to be used for computing similarity estimates between nonidentical sampling sources using a uniform set of epidemiologically relevant comparators; to our knowledge, this is an approach that has no antecedent. Results from the split graph analysis show general agreement with the conceptual framework and serve to demonstrate the epidemiological hierarchy achieved by the rubric despite these sources sharing many of the same attributes. While our estimation of important attributes by no means encompasses the entirety of Campylobacter epidemiology, an examination of pairwise similarity between sources provided supporting evidence that we have managed to capture enough information with our rubric to describe logical relationships between many Campylobacter sampling sources while maintaining secondary associations where there is underlying epidemiological similarity between sources that are less likely to interact directly. As some of the attributes used in this study have general applicability to other organisms, this approach could be extended to other bacterial infectious disease agents. However, this would require a careful examination of the transmission pathways between reservoirs based on a review of the relevant literature and user knowledge.

A major aim of this study was the development of a method for computing an estimate of the epidemiological similarity between bacterial isolates based on common descriptive metadata contained within sampling records. A unique quantitative summary statistic (ε) that comprised multiple layers of epidemiological data (e.g., source, time, and geography of sampling) was used to estimate the epidemiological similarity of isolates (1 − Δε) in a manner that is consistent, systematic, and scalable to entire databases. In this study, we have used this approach to systematically examine a data set comprised of 654 C. jejuni isolates and show that this approach can be used to derive pairwise epidemiological similarity estimates that are consistent with the underlying sampling metadata, generating similarity values that approach unity on isolates that share a source, location, and date of sampling, as in the case of isolates from a confirmed outbreak of campylobacteriosis.

We have also used this metric to compute the ECC, a reflection of the average epidemiological similarity of isolates sharing a molecular subtype.

Calculating the ECC provides an avenue for assessing the performance of a subtyping method based on epidemiological concordance that can be performed independently of other typing methods; our proposed approach also allows the systematic examination of the epidemiological relevance of individual clusters generated by any molecular typing method.

A key driver in the development of novel molecular typing methods is higher discriminatory power. By assigning isolates into smaller clusters, methods with higher discriminatory power are expected to reduce the likelihood that nonepidemiologically related isolates will share the same subtype, thus improving epidemiological concordance. In previous work, we showed that CGF provides higher discriminatory power than MLST while maintaining high concordance with group memberships established by the MLST method (6, 34). By subjecting our data set of 654 C. jejuni isolates to both MLST and CGF and comparing the ECC values of clusters generated by subtyping methods with increasing resolution (i.e., CC < ST < CGF), we aimed to test the hypothesis that strain typing methods with higher resolution would separate isolates of C. jejuni into clusters demonstrating higher epidemiological concordance. Our results indicate the ability of CGF and ST to resolve large clusters produced by using CCs into smaller, more refined clusters with greater epidemiological concordance, as indicated by a higher overall ECC value. It is important to note that although clusters with ECC values approaching unity might appear to be optimal, they necessarily represent groups of isolates with singular temporal, geospatial, and source signals. In the context of infectious disease epidemiology, however, ECC values that deviate significantly from unity are expected due to the transmission and survival of subtypes across a wide range of sampling dates, locations, and sources.

An inherent strength of our model is the flexibility afforded in the inputs for γ, τ, and σ, which can be used to modify the contribution accorded to geospatial, temporal, and source components, respectively. Adjusting the coefficients in the calculation of Δε can be used to limit the signal resulting from unreliable or incomplete data but should also allow more targeted analyses, such as facilitating the identification of subtypes with above-average source, temporal, or geospatial associations. In our original analysis, we combined source, temporal, and geospatial distances using a 50:30:20 ratio, respectively, in order to achieve results that reflected all three components of the model, with nonequal weighting of the coefficients to reflect the importance of source for C. jejuni epidemiology and to reflect the decreased granularity of geospatial data in our data set. When the ECC was recalculated with heavily adjusted σ, τ, and γ percentages favoring source or temporal associations, the overall ECC value decreased when the temporal signal was emphasized, consistent with the wide temporal range spanned by the isolates in the data set (2004 to 2012). An analysis of the outliers revealed certain CGF subtypes that produced very high ECC values when considering the source or temporal signal as the primary metric for evaluation. Thus, by modifying the contribution of the various parameters in our model to Δε, it is possible to adjust the resulting ECC estimates. In a point source outbreak investigation, for example, it may be more suitable to negate the source component of the model entirely, in favor of high temporal and geospatial similarities; this would emphasize groups of isolates collected together in the same place and time, potentially allowing the identification of nonhuman sources of exposure sampled during the time course of a confirmed outbreak. In contrast, adjusting the coefficients to favor source or geospatial relationships could be better suited to performing source attribution or for the identification of pathogens endemic to particular geographic regions, respectively.

Recently, technologies for evaluating the WGSs of bacterial isolates have become widely available, and it is likely that the increasing adoption of WGS will result in the concomitant phasing out of molecular typing methods in the near future. Analysis of WGS data offers unparalleled discriminatory power for comparing bacterial isolates while also providing a wide range of analytical options (e.g., analysis of single nucleotide polymorphisms, gene-by-gene sequence-based typing, and gene content analysis) that facilitate *in silico* comparisons with legacy data sets (28, 35). Furthermore, WGS-based analysis has become sufficiently cost-effective to allow an increasing number of public health laboratories to focus their efforts on the generation of WGSs for isolates collected through routine surveillance (36), resulting in an explosive growth in the number of isolates being analyzed and the concomitant phasing out of molecular typing methods in the very near future. In this context, the potential utility of the framework proposed here resides in the scalability of a scriptable, systematic approach that allows efficient and automatable computation of epidemiological signals, epidemiological similarity, and epidemiological concordance across very large data sets and the flexibility to support different epidemiological applications.

By facilitating the direct comparison of genomic information on bacterial isolates with their underlying epidemiology, our framework provides an epidemiological basis for systematically assessing and interpreting the results obtained from both molecular and WGS-based analyses, which will help improve the optimization of novel genomic approaches in the emerging field of genomic epidemiology.

### Conclusions.

In the rapidly evolving field of molecular epidemiology, improved measures for assessing the genetic similarity of bacterial isolates need to be balanced with equally improved measures for assessing strain epidemiology that allow direct comparisons between the two. Here we have presented a simple model for the quantitative assessment of similarities of human bacterial pathogens based on a comparison of their descriptive sampling attributes. Using a test data set of Canadian C. jejuni isolates spanning a wide range of sampling sources, times, and locations, we have demonstrated that deriving interstrain relationships based on basic epidemiological metadata results in highly structured groups of isolates that conform to a natural, cogent organization. Moreover, by transforming a set of descriptive qualifiers into a quantitative epidemiological summary, we show that this metric can be used toward assessing the epidemiological relevance of subtyping methods as a means of systematically evaluating subtyping method performance.

## MATERIALS AND METHODS

### Description of the EpiQuant model for computing Δε.

The geography of a sample from which a bacterial isolate was recovered, the time or date of sampling, and the source of a sample (i.e., the specific reservoir or vehicle) represent three common metadata descriptors that can be used for broadly describing the ecological epidemiology (i.e., the “ecological address”) of a bacterial isolate. In our model, the “epidemiological type” (ε) of a bacterial isolate is described by its position in a three-dimensional space defined by geospatial (*g*), temporal (*t*), and source (*s*) variables and is thus expressed by the vector

(1) ε=(g, t, s)

A calculation of the “epidemiological distance” between any two isolates can then be defined by a combination of these three distances. A formula expressing the Euclidean distance between the respective vectors is therefore represented by

(2) $$\Delta \varepsilon \;=\;\sqrt{\gamma (\Delta g{)}^{2}\;+\;\tau (\Delta t{)}^{2}\;+\;\sigma (\Delta s{)}^{2}}$$

where Δ*g*, Δ*t*, and Δ*s* represent the pairwise geospatial, temporal, and source distances between the sampling parameters of two isolates and γ, τ, and σ represent adjustable coefficients for assigning relative contributions to each component based on *a priori* considerations of data granularity, reliability, or importance. Substituting derivations for Δ*g*, Δ*t*, and Δ*s* into equation 2 yields our final model for summarizing the epidemiological distance between any two bacterial isolates:

(3) $$\Delta \varepsilon \;=\;\sqrt{\gamma ((\log\{{dist}_{ab}\}{)}^{2})\;+\;\tau (\log\;\{\sqrt{\overset{n}{\underset{i\;=\;1}{\sum }}(x_{i}-y_{i}{)}^{2}}\}{)}^{2}\;+\;\sigma (1\;-\;\frac{1}{n}(\overset{n}{\underset{i\;=\;1}{\sum }}f(u_{i},\nu_{i})){)}^{2}}$$

where *dist_ab_* is the physical distance, in kilometers, between sampling locations for each isolate; *x* and *y* represent the sampling time of each pair of isolates, rounded to the nearest day; and *f*(*v_i_*, *u_i_*) is a function for comparing sampling sources in a conceptual model describing the transmission of C. jejuni (Fig. 1) using a set of epidemiological attributes and the scoring rubric used to compare them (Fig. 2). Finally, the epidemiological similarity between two isolates is defined as 1 − the epidemiological distance (i.e., 1 − Δε). A detailed rationale and derivation of the various components in the complete model are presented in Text S1 in the supplemental material.

### Strain selection for assessing the EpiQuant model.

The majority (*n* = 490) of Campylobacter jejuni isolates included in this study were described previously (6, 34). These isolates were sampled from a wide range of agricultural, environmental, retail, and human clinical sources by the FoodNet Canada enteric disease surveillance network (formerly C-EnterNet) and analyzed by using CGF (6) and MLST (37). Additional C. jejuni isolates were added to this study so as to cover a wider range of geospatial, temporal, and source parameters. These included further isolates collected by FoodNet Canada (*n* = 42) as well as those collected as part of various sampling initiatives from southern Alberta, British Columbia, Ontario, Quebec, and New Brunswick, Canada (*n* = 122). All additional isolates were selected from the Canadian Campylobacter Comparative Genomic Fingerprinting Database (C3GFdb) on the basis of their CGF fingerprint and sampling metadata. The C3GFdb is a pan-Canadian collection of over 22,000 Campylobacter isolates from human clinical, animal, and environmental sources analyzed by CGF.

### DNA extraction and whole-genome sequencing.

Whole-genome sequencing was performed on the isolates used to supplement our original data set (*n* = 164) in order to derive *in silico* MLST profiles. Isolates were recovered from archival glycerol stocks (60% glycerol in phosphate-buffered saline stored at −80°C). Stocks were streaked for isolation onto modified cefoperazone charcoal deoxycholate agar (mCCDA) (CM0739, with selective supplement SR0155E; Oxoid), and monocultures were incubated for 24 to 48 h in a tri-gas microaerobic environment (MAE) (10% CO_2_, 5% O_2_, 85% N_2_) at 42°C. Single colonies were selected and spread onto blood agar plates (BBL blood agar base [catalog number 211037; BD], 5% sheep blood) and incubated overnight in a MAE prior to harvesting of biomass. Genomic DNA extractions were performed by using the Qiagen genomic tip 20G kit according to the manufacturer's recommendations. The quantity and integrity of genomic DNA were assessed by using the Quant-IT HS fluorometric assay (catalog number Q-33120; Life Technologies) and gel electrophoresis on 0.8% agarose, respectively.

Paired-end tagged libraries were prepared at the National Microbiology Laboratory (Winnipeg, Manitoba, Canada) and sequenced on the Illumina MiSeq platform using 150-bp reads. Approximately 30 isolates were pooled per run, yielding, on average, 80- to 100-fold coverage per isolate. Draft genome assemblies were assembled *de novo* by using the St. Petersburg Academy genome assembler (SPAdes version 3.5.0) (38) and selecting a k-mer length of 55, as this provided a consistent quality of assemblies across the data set.

### *In silico* typing of draft genome assemblies.

In order to derive molecular typing results from the WGS data, Microbial *In silico* Typing (MIST) software was used (39). Developed by our group, MIST is an analytical typing engine that enables the user to simulate molecular subtyping results based on a series of user-defined sequence homology searches against draft genome sequence assemblies. For the generation of *in silico* MLST results, we subjected our collection of draft genome assemblies to sequence queries using MLST allelic sequences available from the BIGSdb server, hosted at the Campylobacter PubMLST website (http://pubmlst.org/campylobacter/) (40). CCs and STs were determined based on assignments from PubMLST. A small number of isolates (*n* = 21) had novel alleles and were excluded from ST-based analyses.

### Application of the EpiQuant model framework to isolates of C. jejuni.

All calculations used in the analyses for this study were performed in the R environment for statistical computing (41) using a set of custom scripts available for download (see http://www.github.com/hetmanb/EpiQuant_Typing_Analysis). Pairwise distance matrices for Δ*g*, Δ*t*, and Δ*s* were combined as described in equation 3 to yield a final Δε matrix for all isolates used in the study. To facilitate the exploration of the EpiQuant framework, including various parameters used to calculate the Δε statistic, an interactive Web application was developed by using the R Shiny Web application framework (version 0.14.2.9000) for R (http://shiny.rstudio.com/), available for download (see https://github.com/hetmanb/EpiQuant). A live demonstration of the site is also available (see https://lfz.corefacility.ca/shiny/EpiQuant/).

A two-dimensional neighbor network was generated from a matrix of source distances using the “neighborNet” function from the “phangorn” package (version 2.1.1) in R (42) and edited for visual clarity by using the SplitsTree4 program (version 4.14.3) (43). Heat maps were generated in R by using the heatmap.2 function from the Gplots package (version 3.0.1) (44) and applying single-linkage clustering. The geospatial component of the data set had partial data (i.e., defined at the level of province only) for 63 entries; we assessed these locations as a general provincial location based on Google Maps GPS data (e.g., “Ontario, Canada”).

### Assessing the epidemiological relevance of C. jejuni subtyping data.

We defined the ECC of subtyping clusters as the mean pairwise epidemiological similarity for all isolates within a subtype cluster. The ECC statistic was used as a measure of the epidemiological concordance (i.e., the epidemiological relevance) of subtyping clusters, with a high ECC representing clusters with increased epidemiological specificity (i.e., sampled from similar times, locations, and sources) and a low ECC representing groups of isolates sharing the same subtype despite various epidemiological profiles. Singleton clusters (e.g., clusters containing only one isolate) were not included in the ECC analysis but were used to compute the background ECC signal of nonclustered isolates for use as a basis for comparison to the ECC values of various subtyping clusters. Group comparisons for ECC values of isolates were performed in R (version 3.3.1) using an analysis of variance (“aov”) with follow-up Tukey honestly significant difference testing (“TukeyHSD”), and all tests were performed at a level of significance of an α value of 0.05. To identify outliers from a box plot analysis of CGF subtyping data, we performed a typical Tukey outlier analysis, where subtype clusters with ECC > Q3 + (1.5 × IQR or ECC < Q1 − (1.5 × IQR) were determined to be statistical outliers (where Q1 and Q3 are the first and third quartiles, respectively, and interquartile range [IQR] = Q3 − Q1).

## Supplementary Material

Supplemental material
