# Supplementary material for: The EpiQuant Framework for Computing Epidemiological Concordance of Microbial Subtyping Data
Source: J Clin Microbiol. 2017 Apr 25;55(5):1334–49. doi: 10.1128/JCM.01945-16 (PMC5405252; doi:10.1128/JCM.01945-16)
Supplement: Supplemental material [file supp_55_5_1334__index.html]

The EpiQuant Framework for Computing Epidemiological Concordance of Microbial Subtyping Data — Supplemental material 

# The EpiQuant Framework for Computing Epidemiological Concordance of Microbial Subtyping Data

## Supplemental material

- Supplemental file 1 -

  Text S1 (Description of EpiQuant model derivation)

  PDF, 712K
