# Supplementary material for: The EpiQuant Framework for Computing Epidemiological Concordance of Microbial Subtyping Data
Source: J Clin Microbiol. 2017 Apr 25;55(5):1334–49. doi: 10.1128/JCM.01945-16 (PMC5405252; doi:10.1128/JCM.01945-16)
Supplement: Supplemental material [file JCM.01945-16_zjm999095448s1.pdf]

Description of EpiQuant Framework

1. Development of the model framework

The geography of a sample from which a bacterial isolate was recovered, the time or date of sampling, and the source of the sample, represent three common metadata descriptors that can be used for uniquely describing the epidemiology of a bacterial isolate. Much like a sequence of genetic features can be used to create a strain genotype in molecular epidemiology, this combination of descriptive epidemiological parameters can be used to describe the epidemiologic type or *epi-type* of an isolate, which can thus be used to assess the epidemiologic similarity of any two bacterial isolates collected in a surveillance setting. Our aim is thus to develop an approach that can be used to compute the epidemiological distance between two isolates based on a quantitative comparison of epi-types.

In our model, the epidemiologic type or *epi-type* ($\mathcal{E}$) of a bacterial isolate can be described by its position in a three-dimensional space defined by geospatial (g), temporal (t), and source (s) components and defined by the vector:

$$\varepsilon = (g, t, s) \tag{1}$$

The *Epidemiological Distance* between two epi-types ($\Delta\mathcal{E}$) is given by the weighted Euclidean distance between their respective vectors:

$$\Delta\varepsilon = \sqrt{\gamma(\Delta\, g)^2 + \tau(\Delta\, t)^2 + \sigma(\Delta\, s)^2} \tag{2}$$

where Δg, Δt, and Δs represent the pairwise geospatial, temporal and source distances respectively and γ, τ, and σ represent adjustable coefficients for assigning weights to each component based on *a priori* epidemiological considerations. For example, a bacterial species known to be highly source-restricted may then require higher value for σ to provide additional weight to the source relative to the geospatial and temporal variables, to account for the increased significance when observing a difference in the source.

2. Defining the components of the model.

Since each component of the $\mathcal{E}$ vector represents a different form of information (geospatial, temporal, source), the distance calculation in each dimension requires a different mathematical treatment.

A) Geospatial distance (Δg):

The geographical distance between pairs of isolates is computed based on geographical positioning system (GPS) coordinates with distances between GPS coordinates calculated using geog.dist function of the 'fossil' package in R (1). Thus, the equation for calculating the geographic distance between two bacterial isolates can be written as:

$$\gamma(\Delta g) = \gamma(\{dist_{ab}\}) \quad (3)$$

where ($dist_{ab}$) is the physical distance, in kilometres, between each pairwise comparison of isolates, calculated using the Haversine formula for deriving great-circle spherical distances from latitude and longitude GPS coordinates (2).

B) Temporal distance (Δt):

The temporal distance between pairs of isolates is computed based on the formula:

$$\tau(\Delta t) = \tau(\{\sqrt{\sum_{i=1}^{n}(x_i - y_i)^2}\}) \quad (4)$$

where (x, y) represent the date of isolation of each pairing of isolates, in POSIX-time, which is defined as the time elapsed since January 01, 1970, rounded to the nearest day.

C) Source distance (Δs):

The source component is inherently more complex to quantify and to our knowledge, no system currently exists for estimating the likeliness of one epidemiologic source compared to another. Approaches based on using the genetic similarity of sources may provide good basis for assessing the similarity of plant or animal sources, however, when comparing environmental samples such as water or soil, this method loses its effectiveness. Because our example in this study uses data for *Campylobacter jejuni*, we chose to employ categories commonly used in describing the epidemiology of enteric pathogens (3). To this end, sources were redefined as fitting to animal, human or environmental categories, and then further differentiated based on additional epidemiologic attributes pertaining to each parental category. In essence, a line-list was created containing all the non-redundant sources in the dataset as the sample input, with descriptive epidemiologic attributes acting as the informative elements of the questionnaire. Each source exemplar is then assessed independently across all attributes with three possible outcomes for each attribute: strong association, partial or potential association, and little to no association. This effectively reduces each source into a consistent set of comparable attributes, which allows us to compute the distance for pairs of sources (Δ*s*) based on the matching and partially matching attributes as a proportion of the total number of attributes examined. Thus, the statistic for source distance becomes:

$$\sigma(\Delta s) = \sigma\left(1 - \frac{1}{n}\left(\sum_{i=1}^{n} f(u_i, v_i)\right)\right) \quad (5)$$

where $f(v_i, u_i)$ is the function to compute the pairwise source similarity score from a matrix comprising rows of each source and columns of defined epidemiological attributes. The function $f(v_i, u_i)$ compares

the score from sources *u, v* in the column position *i* and based on complete, partial, or negative matches, returns a predefined score:

- (0-0) matches: score of 1
- (* - *) partial matches: score of 0.8
- (*-0), (0-*), (1-*) and (*-1) partial matches: score of 0.2
- (1-0) and (0-1) mismatches: score of 0.

The sum of scores across all attributes is then divided by the total number of attributes resulting in a pairwise similarity estimate for two sources normalized to 1. Using this approach, it becomes possible to assign a pairwise similarity to any two bacterial isolates based solely on their descriptive epidemiologic source.

3. Derivation of the Δ*E* statistic.

To account for the skewed contributions of geospatial and temporal components when Δg and Δt are high (4), we apply a logarithmic correction to the distribution of these data in the dataset. Our rationale is that the epidemiological signal of geographical and temporal distances should decay rapidly as these distances increase. The epidemiological relevance of temporal information for isolates separated by 1 year should have no greater impact than that of isolates separated by 10 years and we expect a similar relationship for geographical distance. Conversely, for isolates sampled with very close geographical or temporal proximity, the epidemiological significance of geographical or temporal data is likely to be extremely high. Thus, by applying a logarithmic correction to Δg and Δt, we shape the distribution of the resulting similarity values such that they provide a greater significance to isolates of closer temporal or geographic distance.

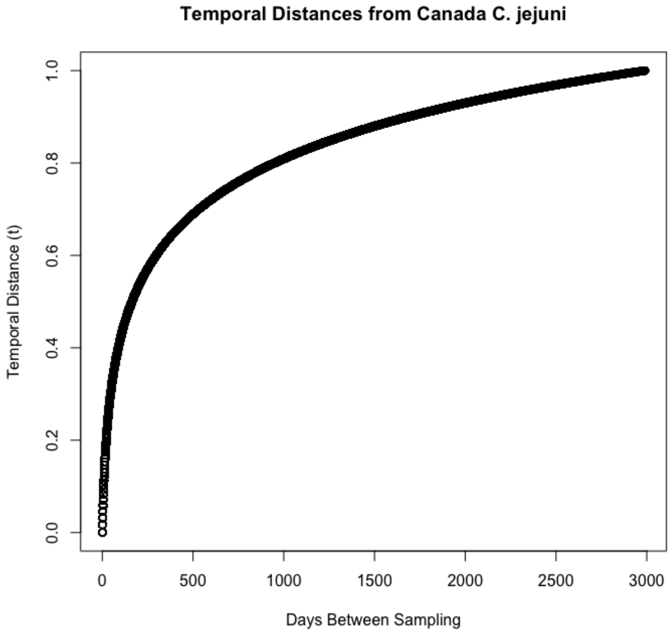

Figure 1: Temporal distance calculated from sampling metadata accompanying 654 isolates of Canadian *C. jejuni*

To calculate the relative geospatial distance for pairs of isolates in the dataset the distances (in km) are calculated between each isolate pair and treated as a proportion of the largest distance in the dataset. A similar treatment is performed on the temporal data, where the individual pairwise distances (in number of days) are calculated based on the day of isolation of each isolate and treated as a proportion of the largest temporal distance in the dataset. This effectively reduces each estimate to a proportional value out of 1, and allows us add the contributions from geospatial distance, temporal distance and source distance together directly.

Substituting the geospatial, temporal and source distance equations (3 – 5) and incorporating the logarithmic corrections into Equation 2 yields our final model for computing the basic *Epidemiologic Distance* metric ($\Delta\mathcal{E}$) between any two bacterial isolates, which is presented in Equation 6:

$$\Delta\varepsilon = \sqrt{\gamma(\log\{dist_{ab}\})^2 + \tau\left(\log\left\{\sqrt{\sum_{i=1}^{n}(x_i - y_i)^2}\right\}\right)^2 + \sigma\left(1 - \frac{1}{n}\left(\sum_{i=1}^{n} f(u_i, v_i)\right)\right)^2} \tag{6}$$

References:

1. **Vavrek MJ**. 2012. Fossil: palaeoecological and palaeogeographical analysis tools. Palaeontol Electron http//palaeo-electronicaorg/2011_1/238/indexhtml R Packag version 030.

2. **Sinnot RW**. 1984. Virtues of the Haversine. Sky Telescope **68**:158–159.

3. **Harding S**, **Parmley J**, **Morrison K**. 2014. Using participatory epidemiology to assess factors contributing to common enteric pathogens in Ontario: results from a workshop held at the Ontario Veterinary College, University of Guelph, Ontario. BMC Public Health **14**:405.

4. **French N**, **Barrigas M**, **Brown P**, **Ribiero P**, **Williams N**, **Leatherbarrow H**, **Birtles R**, **Bolton E**, **Fearnhead P**, **Fox A**. 2005. Spatial epidemiology and natural population structure of Campylobacter jejuni colonizing a farmland ecosystem. Environ Microbiol **7**:1116–1126.
